# Meta-Analysis of HER2-Enriched Subtype Predicting the Pathological Complete Response Within HER2-Positive Breast Cancer in Patients Who Received Neoadjuvant Treatment

**DOI:** 10.3389/fonc.2021.632357

**Published:** 2021-07-23

**Authors:** Guoshuang Shen, Fuxing Zhao, Xingfa Huo, Dengfeng Ren, Feng Du, Fangchao Zheng, Jiuda Zhao

**Affiliations:** ^1^ Breast Disease Diagnosis and Treatment Center of Affiliated Hospital of Qinghai University & Affiliated Cancer Hospital of Qinghai University, Xining, China; ^2^ Key Laboratory of Carcinogenesis and Translational Research, The VIPII Gastrointestinal Cancer Division of Medical Department, Peking University Cancer Hospital and Institute, Beijing, China; ^3^ Department of Medical Oncology, Cancer Hospital, Chinese Academy of Medical Sciences, Peking Union Medical College, Beijing, China

**Keywords:** human epidermal growth factor receptor 2 (HER2)-enriched subtype, HER2-postive breast cancer, neoadjuvant treatment, pathologic complete response, meta-analysis

## Abstract

**Background:**

This meta-analysis aimed to better elucidate the predictive value of human epidermal growth factor receptor 2 (HER2)-enriched subtype of pathological complete response (pCR) rate within HER2-positive breast cancer patients receiving neoadjuvant treatment.

**Methods:**

We identified prospective trials that evaluated the correlation between an HER2-enriched subtype and pCR rate in HER2-positive breast cancer. Pooled odds ratio (OR) values with 95% confidence intervals (CIs) were computed.

**Results:**

Fifteen studies comprising 2,190 patients met the inclusion criteria. The HER2-enriched subtype was associated with increased odds of achieving a pCR (OR = 4.12, 95% CI = 3.38 to 5.03, *P* < 0.001) in patients overall. Moreover, it was correlated with improved pCR when single or dual HER2-targeted agent-based therapy was employed (OR = 3.36, 95% CI = 2.25 to 5.02, *P* < 0.001; OR = 4.66, 95% CI = 3.56 to 6.10, *P* < 0.001, respectively), but not when HER2-targeted agent-free chemotherapy was used (OR = 2.52, 95% CI = 0.98 to 6.49, P = 0.05). Moreover, an HER2-enriched subtype predicted higher pCR rates irrespective of HER2-targeted agents (trastuzumab, lapatinib, pertuzumab, or T-DM1); chemotherapy agents (taxane-based, or anthracyclines plus taxane-based); endocrine therapy and hormone receptor [all the differences were statistically significant (*P* all ≤ 0.001)].

**Conclusions:**

The HER2-enriched subtype can more effectively and specifically predict pCR for HER2-targeted agent-based neoadjuvant treatment, irrespective of the number (single or dual) or category of HER2-targeted agent, including chemotherapy and endocrine therapy, or hormone receptor in cases of HER2-positive breast cancer.

## Introduction

Neoadjuvant treatment can induce tumor downstaging in locally advanced breast cancer and enable surgical tumor removal. In addition, this therapy can increase rates of breast conservation operation in women who would otherwise need a mastectomy (1). Amplification or over-expression of human epidermal growth factor receptor 2 (HER2), which is observed in approximately 20% of breast cancers, is often associated with highly aggressive tumor behavior and poor outcomes (2). Within a neoadjuvant treatment setting, HER2-positive patients encompass up to ~30% of total patients (3). Fortunately, neoadjuvant treatment, especially when chemotherapy is combined with HER2-targeted agents, is extremely effective for combating HER2-positive breast cancer. To date, chemotherapy plus HER2-targeted therapy may achieve ≥60% of pathological complete response (pCR, defined as ypT0/is or ypT0/is ypN0) rates (4). In addition, a more recent meta-analysis comprised of more than 5,500 patients with HER2-positive breast cancer showed that pCR is associated with improved prognoses (5).

However, although patients with HER2-positive breast cancer are likely to respond to neoadjuvant treatment with HER2-targeted agent-based regimens, there remain some patients that benefit less from the treatment, as HER2-positive breast cancer exhibits high biological heterogeneity (6–8). Therefore, only some patients are able to achieve pCR, while others that only show a partial response or stable disease even exhibit progress. Therefore, it is essential to establish the molecular determinants of response-associated biomarkers that can enable identification of patients who may benefit from HER2-targeted agent-based neoadjuvant treatment, and subsequently implement personalized therapy. Neoadjuvant clinical treatment studies have found that the most significant predictors of treatment response include the following: expression levels of HER2; the HER2-enriched subtype; high levels of tumor-infiltrating lymphocytes (TILs); the estrogen receptor gene ESR1; as well as mutations in phosphatase phosphoinositol-3 (PI3) kinase (PIK3CA), genes related to gene expression signatures, pathways, and mutation profiles (9). Among them, the HER2-enriched subtype is one of the most frequently reported predictors in the analyses of prospective neoadjuvant trials, such as NeoALTTO, CALGB40601, NOAH, and CherLOB (8, 10–12).

Different from conventional typing of breast cancer that is based on four protein biomarkers including estrogen receptor (ER), progesterone receptor (PR), HER2, and Ki-67, intrinsic molecular subtype of breast cancer is analyzed through gene expression profiling which includes 50 genes. Intrinsic molecular subtype of HER2-positive breast cancer includes luminal A, luminal B, HER2-enriched, and basal-like and can better represent the inherent biologic heterogeneity of breast cancer (13, 14); we define luminal A, luminal B, and basal-like as no-HER2-enriched subtype. The HER2-enriched subtype accounts for ~50–60% of the intrinsic molecular subtypes of HER2-positive breast cancer (13, 14). Compared with the other subtypes, those of the HER2-enriched subtype have a higher likelihood of achieving a pCR following anti-HER2-based treatment, as demonstrated in several trials (8, 10–12). Nevertheless, most published studies only employed relatively small sample sizes and involved diverse treatment regimens, and the results were inconsistent. Thus, there remain no confirmed nor concurrent results concerning the value of the HER2-enriched subtype in predicting the pCR rate in neoadjuvant treatment within HER2-positive breast cancer. Therefore, the meta-analysis presented here analyzing published prospective clinical trials aimed to establish pooled estimates for pCR based on the presence of the HER2-enriched subtype in patients with HER2-positive breast cancer that received neoadjuvant treatment.

## Methods

### Literature Search

A systematic review of the literature and meta-analysis was performed to identify prospective controlled or single-arm trials that explored the association between the presence of the HER2-enriched subtype in patients with HER2-positive breast cancer and pCR rate in a neoadjuvant treatment setting. A prerequisite for the query was that HER2-positive breast cancer was identified based on intrinsic subtypes. This analysis was conducted following the preferred reporting items for systematic reviews and meta-analyses (PRISMA) statement (15).

Inclusion criteria for this meta-analysis were as follows: (1) patients must have been diagnosed with HER2-positive breast cancer and be recipients of neoadjuvant treatment; (2) included studies evaluated the relationship between the HER2-enriched subtype and pCR (defined as ypT0/is or ypT0/is ypN0) rate; (3) intrinsic molecular subtypes were present at baseline; (4) studies must have been either prospective randomized controlled or single-arm trials; (5) adequate data needed to be provided in order to estimate the odds ratio (OR) for pCR for each HER2 subgroup (*i.e.* HER2-enriched *versus* non-HER2-enriched); and (6) all original research articles must have been published in English. When two or more studies were reported by the same authors, the higher quality research manuscript was included. The following studies were excluded: (1) overlapping studies or those containing duplicate data; (2) reviews, comments, letters, or animal studies; (3) studies published in non-English languages; and (4) retrospective studies.

Two independent reviewers queried PubMed/MEDLINE, Scopus, Cochrane Library, EMBASE, and ClinicalTrials.gov and identified articles published up until November 2019. In addition, the American Society of Clinical Oncology (ASCO), European Society for Medical Oncology (ESMO), San Antonio Breast Cancer Symposium (SABCS), and American Association for Cancer Research (AACR) websites were searched for relevant presentations and abstracts. Search terms included: “breast cancer”, “breast neoplasms”, “breast tumor”, “breast carcinoma”, “HER2-positive”, “human epidermal growth factor receptor 2-positive”, “HER2-enriched”, “HER2-E”, “intrinsic subtypes”, “PAM50”, “neoadjuvant”, and “preoperative”.

### Data Extraction

Two reviewers (GS and FuZ) independently extracted the following data: first author, year of publication, study design, number of patients enrolled, neoadjuvant treatment regimens, pCR definition and rates in the original trials, pCR rates within HER2-enriched and non-HER2-enriched subgroups (*i.e.* luminal A, luminal B, basal-like, and normal-like), and hormone receptor status.

### Data Synthesis and Statistical Analysis

The primary endpoint was to investigate the association between the HER2-enriched subtype and pCR rates within HER2-positive breast cancer patients having received neoadjuvant treatment with HER2-targeted agents either combined with or without chemotherapy or endocrine therapy.

The secondary objective was to evaluate the association between an HER2-enriched subtype and pCR rates according to number and category of employed HER2-targeted agents, chemotherapy, endocrine therapy, and hormone receptor status. Three groups were identified according to the HER2-targeted agent administered: single HER2-targeted agent, dual HER2-targeted agents, and HER2-targeted agent free. Four additional groups were identified according to the HER2-targeted agent used: trastuzumab, pertuzumab, lapatinib, or trastuzumab emtansine (T-DM1). Two chemotherapy groups were also considered: taxane-based and anthracyclines plus taxane-based. In addition, three groups were analyzed based on endocrine therapy, and one group was analyzed based on hormone receptor status.

Patients exhibiting pCR in each of the HER2-enriched subtype subgroups of the overall study population were confirmed and then categorized by different treatment regimens. We adopt the definition of pCR of the primary objective if the definition of pCR for the primary and secondary objectives differed within a study. Data were expressed using OR and the corresponding 95% confidence intervals (CIs) using two-sided *P*-values. The OR extracted from each study provided an estimate of the ratio of the pCR rate for HER2-enriched *vs.* non-HER2-enriched subtypes. Then subgroup analyses were performed according to the treatment regimens. Fixed-effects models (Mantel–Haenszel, *P* > 0.1 and *I^2^* < 50%) assumed that differences between the results of different studies were due to chance. Significant heterogeneity was considered to exist when *P <*0.1 or *I^2^ >*50%. When heterogeneity was present, the random-effects model was used, resulting in wider intervals and a more conservative estimate of the effect (16). Within the present meta-analysis, the pooled OR of the pCR was calculated using the fixed-effects models, as no significant heterogeneities were observed in any of the analyses conducted. Sensitivity analyses were performed by recalculating the pooled OR estimates through removal of non-randomized studies (16, 17). Interaction *P*-values of variables were assessed using Chi^2^ statistics. Possible publication bias was assessed using a funnel plot (18, 19). All tests were two-sided, and statistical significance was defined as *P <*0.05. All statistical analyses were performed using Review Manager software version (ver. 5.3, Cochrane Collaboration).

## Results

### Search Results and Characteristics of Eligible Studies

A total of 506 relevant articles were identified in a primary literature search (**Supplementary Figure 1**). After exclusion of duplicate references and those that did not satisfy the inclusion criteria, 15 candidate articles were included in the meta-analysis (8, 10–12, 20–29). Three studies were published only in abstract form (26, 28, 29). All features of the eligible studies in the meta-analysis were summarized in **Table 1**.

**Table 1:** Baseline characteristics of included studies.

Study	Trial Phase	Randomized	Authors	Published years	Neoadjuvant regimen	Number of participants					pCR rate (%)		pCR definition
						HER2-enriched	Non-HER2-enriched				HER2-enriched	Non-HER2-enriched	
							Luminal A	Luminal B	Basal-like	Normal-like			
ACOSOG Z1041 (21)	III	Y	R. Lesurf	2017	FEC→TH *vs*. TH→ FEC	14	28				78.57 (11/14)	39.29 (11/28)	ypT0/is
BERENICE (20)	II	N (Investigator choice)	S.M. Swain	2018	ddAC→TPH	80	33	24	11	NA	75.00 (60/80)	44.12 (30/68)	ypT0/is ypN0
					FEC→DPH	95	31	15	5	NA	73.68 (70/95)	43.14 (22/51)	
CALGB 40601 (8)	III	Y	Carey LA	2015	TH	24	39	32	4	4	70.83 (17/24)	37.97 (30/79)	ypT0/is
					THL	35	30	30	7	0	80.00 (28/35)	40.30 (27/67)	
					TL	23	11	18	3	2	52.17 (12/23)	20.59 (7/34)	
CherLOB (12)	II	Y	Dieci MV	2016	T→FEC+H, L or both	22	21	14	22	NA	50.00 (11/22)	14.03 (8/57)	ypT0/is ypN0
Katsuhiko Nakatsukasa (24)	NA	N	Nakatsukasa K	2016	HER-TC	21	20				47.62 (10/21)	40.00 (8/20)	ypT0/Tis ypN0
KRISTINE (28)	III	Y	Prat A	2018	T-DM1+P	90	35	42	16	NA	62.22 (56/90)	26.88 (25/93)	ypT0/is, ypN0
					TCbH+P	104	25	32	10	NA	72.12 (75/104)	32.84 (22/67)	
MDACC/I-SPY (26)	Not mentioned	Not mentioned	MCU Cheang	2011	AC or FEC or FAC→T	26	6	7	8	NA	46.15 (12/26)	19.05 (4/21)	ypT0/is
NeoALTTO (10)	III	Y	Fumagalli D	2016	H,L or H+L→T	110	58	41	24	21	51.82 (57/110)	21.53 (31/144)	ypT0/is
NOAH (11)	III	Y	Prat A	2014	AT→T→CMF	29	5	7	3	7	27.59 (8/29)	18.18 (4/22)	ypT0/Tis ypN0
					ATH→TH→CMFH	34	7	4	5	13	52.94 (18/34)	34.48 (10/29)	
NSABP B-41 (23)	III	Y	Swain SM	2019	AC→TL	69	26				49.28 (34/69)	34.62 (9/26)	ypT0/Tis ypN0
					AC→TH	71	23				64.79 (46/71)	17.39 (4/23)	
					AC→THL	57	25				70.18 (40/57)	24.00 (6/25)	
Opti-HER HEART (25)	II single-arm	N	Gavilá J	2019	NPLD and TPH	30	6	6	7	9	80.00 (24/30)	46.43 (13/28)	ypT0/Tis ypN0
PAMELA (22)	II single-arm	N	Llombart-Cussac A	2017	HL± letrozole or tamoxifen	101	22	16	9	3	40.59 (41/101)	10.00 (5/50)	ypT0/is
TBCRC006 (27)	II single-arm	N	Prat A	2019	HL± letrozole	73	41				27.40 (20/73)	9.76 (4/41)	ypT0/is
TBCRC023 (29)	II	Y	Prat A	2018	HL± letrozole	51	4	7	11	12	27.45 (14/51)	8.82 (3/34)	ypT0/is
XeNA (26)	II	N	MCU Cheang	2011	HXT	12	2	7	3	NA	58.33 (7/12)	8.33 (1/12)	ypT0/is

There were 45 not applicable, and 61 missing patients of intrinsic subtype in the BERENICE study were not shown in the table; three claudin low subtypes of CALGB 40601 study were not shown in the table. pCR, pathological complete response; ypT0/isypN0, no residual invasive cancer cells in surgical specimens of both primary tumor and axillary lymph node and allowing for ductal carcinoma in situ (DCIS); ypT0/is, no residual invasive cancer cells in surgical specimens of primary tumor regardless of axilla and allowing for DCIS. FEC→TH, fluorouracil, epirubicin, and cyclophosphamide→ paclitaxel and trastuzumab; TH→FEC, paclitaxel and trastuzumab→ fluorouracil, epirubicin, and cyclophosphamide; ddAC→TPH, dose-dense doxorubicin plus cyclophosphamide→paclitaxel and trastuzumab plus pertuzumab; NA, not available; FEC→DPH, fluorouracil, epirubicin, and cyclophosphamide→docetaxel and trastuzumab plus pertuzumab; TH, paclitaxel plus trastuzumab; THL, paclitaxel plus trastuzumab and lapatinib; TL, paclitaxel plus lapatinib; T→FEC+H, L or both, paclitaxel → FEC plus trastuzumab, lapatinib or both; HER-TC, docetaxel, cyclophosphamide, and trastuzumab; T-DM1+P, trastuzumab emtansine and pertuzumab; TCbH+P, docetaxel, carboplatin, trastuzumab, and pertuzumab; AC, doxorubicin and cyclophosphamide; AC or FEC or FAC→T, doxorubicin and cyclophosphamide or 5-fluorouracil, epirubicin and cyclophosphamide or 5-fluorouracil, doxorubicin and cyclophosphamide→paclitaxel; H, L or H+L→T, trastuzumab, lapatinib, or the combination→paclitaxel; AT→T→CMF, doxorubicin and paclitaxel→paclitaxel→cyclophosphamide,methotrexate and fluorouracil; ATH→TH→CMFH, doxorubicin, paclitaxel, and trastuzumab→paclitaxeland trastuzumab→cyclophosphamide, methotrexate, fluorouracil, and trastuzumab; AC→TH, doxorubicin and cyclophosphamide →paclitaxel and trastuzumab; AC→TL, doxorubicin and cyclophosphamide→paclitaxel and lapatinib; AC→THL, doxorubicin and cyclophosphamide→paclitaxel, trastuzumab, and lapatinib; NPLD and TPH, non-pegylated liposomal doxorubicin, paclitaxel,trastuzumab, and pertuzumab; HL, trastuzumab and lapatinib; HXT, capecitabine, docetaxel, and trastuzumab.

These studies evaluated the HER2-enriched subtype and pCR for HER2-positive breast cancer cases in a neoadjuvant treatment setting and consisted of 2,190 participants in total. HER2-targeted agents including trastuzumab, pertuzumab, lapatinib, and T-DM1 were used either alone or within a dual HER2 blockade. The majority of neoadjuvant chemotherapy regimens contain anthracycline and taxanes. The administrated neoadjuvant endocrine agents were letrozole or tamoxifen (TAM).

Of the included studies, eight randomized patients within two or three groups (8, 10–12, 21, 23, 28, 29), six were prospective non-randomized trials (20, 22, 24–27), and one combined two cohorts (26).

### Correlations Between an HER2-Enriched Subtype and pCR Rates in the Overall Study Population

Seven studies defined pCR as having no residual invasive cancer cells within surgical specimens of both the primary tumor and axillary lymph node and allowed for ductal carcinoma *in situ* (DCIS) (ypT0/isypN0) (11, 12, 20, 23–25, 28), while the other eight studies defined pCR as a lack of residual invasive cancer cells within surgical specimens of primary tumors regardless of axilla and allowed for DCIS (ypT0/is) (8, 10, 21, 22, 26, 27, 29).

The pCR rates ranged from 8.3 to 80.0% within the 15 studies, with 27.4–80.0% and 8.3–46.4% within the HER2-enriched and non-HER2-enriched subtypes, respectively.

**Figure 1:**
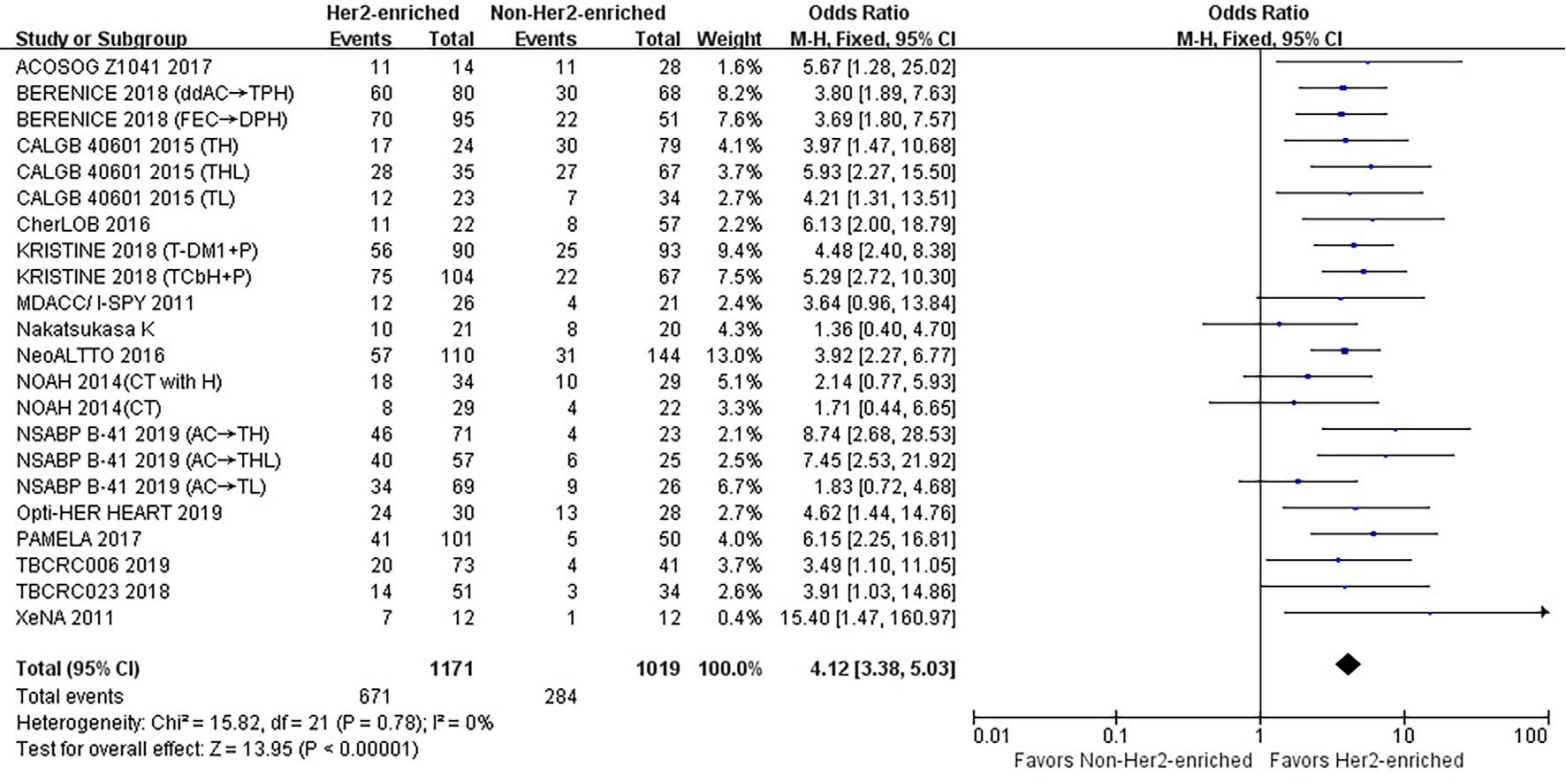
Forrest plot of odds ratio (OR) used to evaluate the correlations between intrinsic molecular subtype subgroups (HER2-enriched *vs.* non-HER2-enriched) and pCR for the overall study population. CI, confidence interval; HER2, human epithelial growth factor receptor 2; pCR, pathological complete response.

**Figure 2:**
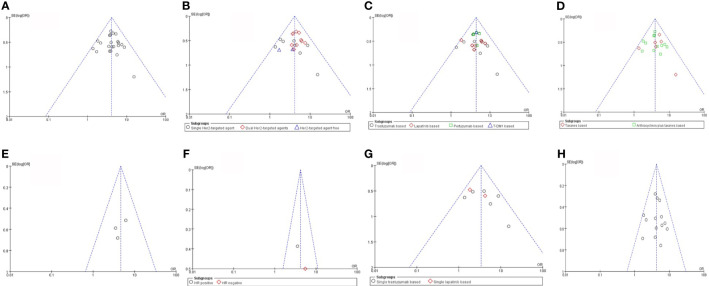
Risk of bias assessment. **(A)** Her2-enriched vs Non-Her2-enriched subtype **(B)** The number of Her2-targeted agents **(C)** The category of Her2-targeted agents **(D)** Different chemotherapy regimens **(E)** Endocrine theraphy **(F)** Hormone receptor **(G)** Trastuzumab alone and Lapatinib alone **(H)** Trastuzumab vs Lapatinib. HER2, human epithelial growth factor receptor 2; HR, hormone receptors status.

Overall the 15 studies contained 2,190 patients, and the HER2-enriched subtype predicted a substantially higher pCR rate (OR = 4.12, 95% CI = 3.38 to 5.03, *P* < 0.001) (**Figure 1**). There was low heterogeneity (*I* ^2^ = 0%, *P* = 0.78) among the studies. A funnel plot shows that there was no evidence of publication bias (**Figure 2**).

There were no correlations observed between either the intrinsic molecular subtype subgroups (HER2-enriched *vs.* non-HER2-enriched subtype) and different numbers of HER2-targeted agents used (*P* = 0.25), the intrinsic molecular subtype subgroups and different categories of HER2-targeted agents (*P* = 1.00), the intrinsic molecular subtype subgroups and type of neoadjuvant chemotherapy regimen (*P* = 0.50), or between the intrinsic molecular subtype subgroups and hormone receptor status (*P* = 0.51).

### Correlations Between HER2-Enriched Subtype and pCR Rates According to the Number of HER2-Targeted Agents Used

#### Single HER2-Targeted Agent

As shown in **Table 1**, eight studies or subgroups compared correlations between an HER2-enriched subtype and pCR in a trial arm encompassing treatment with a single HER2-targeted agent-based therapy (8, 11, 21, 23, 24, 26). These studies included 268 HER2-enriched and 251 non-HER2-enriched patients, with 155 and 80 patients exhibiting pCR within each subtype, respectively. Six studies employed neoadjuvant trastuzumab (11, 21, 24, 26), while two administered neoadjuvant lapatinib as an HER2-targeted therapy (8, 23). The primary chemotherapy regimens were based on anthracyclines and taxanes; four studies used anthracyclines plus taxanes combined with other chemotherapeutics (11, 21, 23), while the other four prescribed either taxanes alone or in combination with other chemotherapy agents (8, 24, 26).

**Figure 3:**
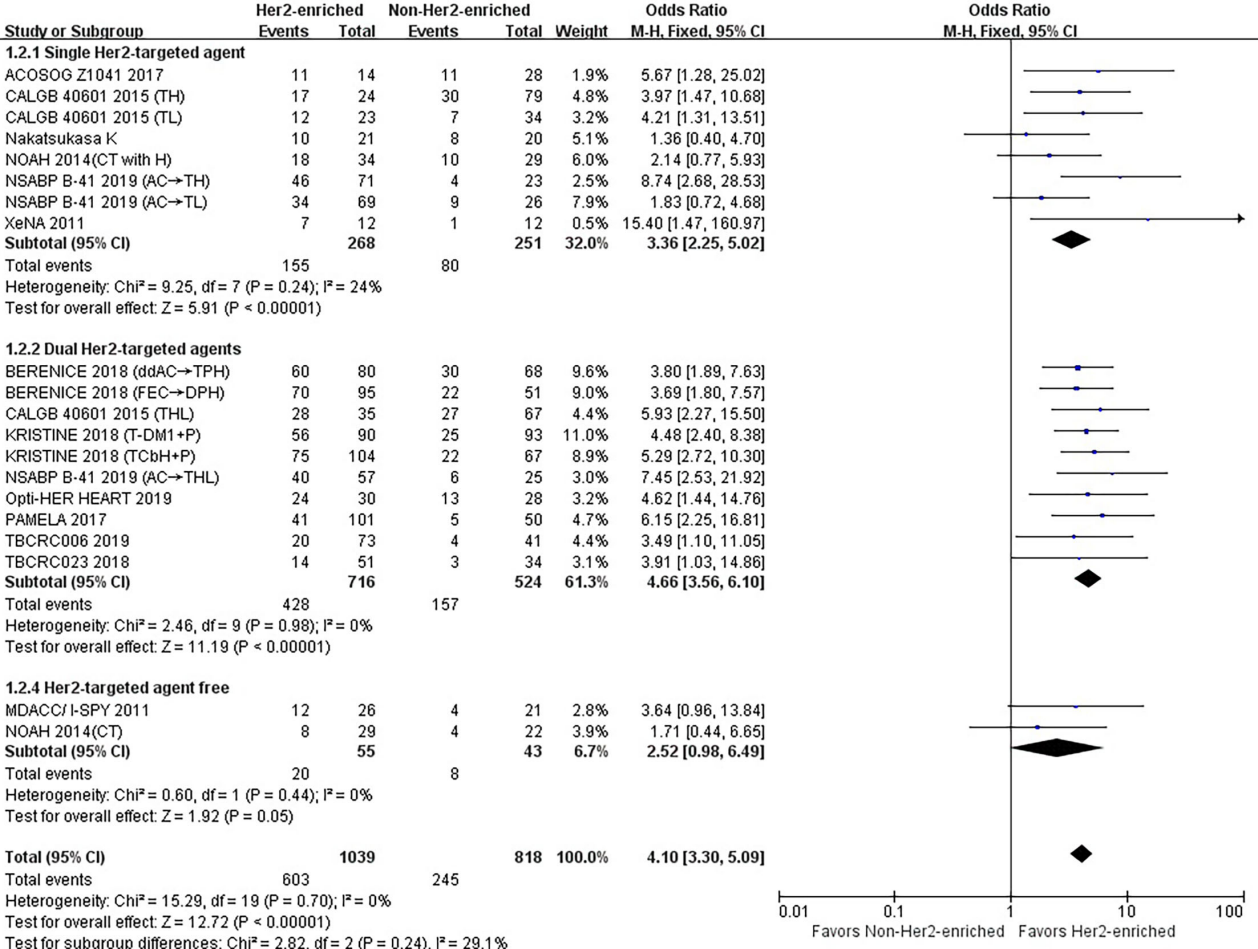
Forrest plot of odds ratio (OR) used to evaluate the correlations between intrinsic molecular subtype subgroups (HER2-enriched *vs.* non-HER2-enriched) and pCR as based on the number of HER2-targeted therapies used. CI, confidence interval; HER2, human epithelial growth factor receptor 2; pCR, pathological complete response.

The results showed that the pCR rate of single HER2-targeted agent treatment for HER2-positive patients was significantly higher for those in the HER2-enriched subgroup than for those in the non-HER2-enriched subgroup (OR = 3.36, 95% CI = 2.25 to 5.00, *P* < 0.001), (*I* ^2^ = 24%, *P* = 0.24) (**Figure 3**).

#### Dual HER2-Targeted Agents

Ten studies or subgroups included 716 patients of the HER2-enriched and 524 of the non-HER2-enriched subgroups, with 428 and 157 patients exhibiting pCR, respectively (8, 20, 22, 23, 25, 27–29). Five studies administered neoadjuvant trastuzumab plus lapatinib (8, 22, 23, 27, 29), four used neoadjuvant trastuzumab plus pertuzumab (20, 25, 28), and one used neoadjuvant pertuzumab plus T-DM1 as HER2-targeted therapy (28). The majority of chemotherapy regimens included anthracyclines and/or taxanes. Four studies used anthracyclines plus taxanes combined with other chemotherapeutics (20, 23, 25), two used only taxanes as chemotherapeutics (8, 28), three employed endocrine agents (letrozole/TAM) (22, 27, 29), while one only used dual HER2-targeted agents (28).

The pooled results demonstrated that the pCR rate of dual HER2-targeted agent treatment was observed significantly higher in the HER2-enriched group (OR = 4.66, 95% CI = 3.56 to 6.10, *P* < 0.001), (*I* ^2^ = 0%, *P* = 0.98) (**Figure 3**).

#### HER2-Targeted Agent-Free

One study and one subgroup, including 55 patients in the HER2-enriched and 43 in the non-HER2-enriched subgroups, included twenty and eight patients exhibiting pCR, respectively (11, 26). Both subgroups administered anthracyclines plus taxanes as chemotherapy regimens.

The combined results showed that the pCR rate of non-HER2-targeted agent treatment in the HER2-enriched group was similar to that in the non-HER2-enriched subtype group (OR = 2.52, 95% CI = 0.98 to 6.49, *P* = 0.05), (*I* ^2^ = 0%, *P* = 0.44) (**Figure 3**).

### Correlations Between HER2-Enriched Subtype and pCR Rates According to the Category of HER2-Targeted-Based Agents

#### Trastuzumab

Fifteen studies or subgroups compared the correlations between the HER2-enriched subtype and pCR in trials including the HER2-targeted agent trastuzumab-based therapy (8, 11, 20–29). These studies included 802 patients within the HER2-enriched and 622 non-HER2-enriched subtypes, with 481 and 196 patients exhibiting pCR, respectively. Six studies used neoadjuvant trastuzumab as a single HER2-targeted therapy (8, 11, 21, 23, 24, 26), five administered neoadjuvant trastuzumab combined with lapatinib as dual HER2-targeted therapy (8, 22, 23, 27, 29), and four used neoadjuvant trastuzumab combined with pertuzumab as dual therapy (20, 25, 28). The primary chemotherapy regimens were based on anthracyclines and taxanes. Anthracyclines plus taxanes or in combination with other chemotherapeutics, or taxanes alone were administered in seven (11, 20, 21, 23, 25) and five (8, 24, 26, 28) studies, respectively. In addition, three studies used letrozole or TAM as endocrine treatment.

**Figure 4:**
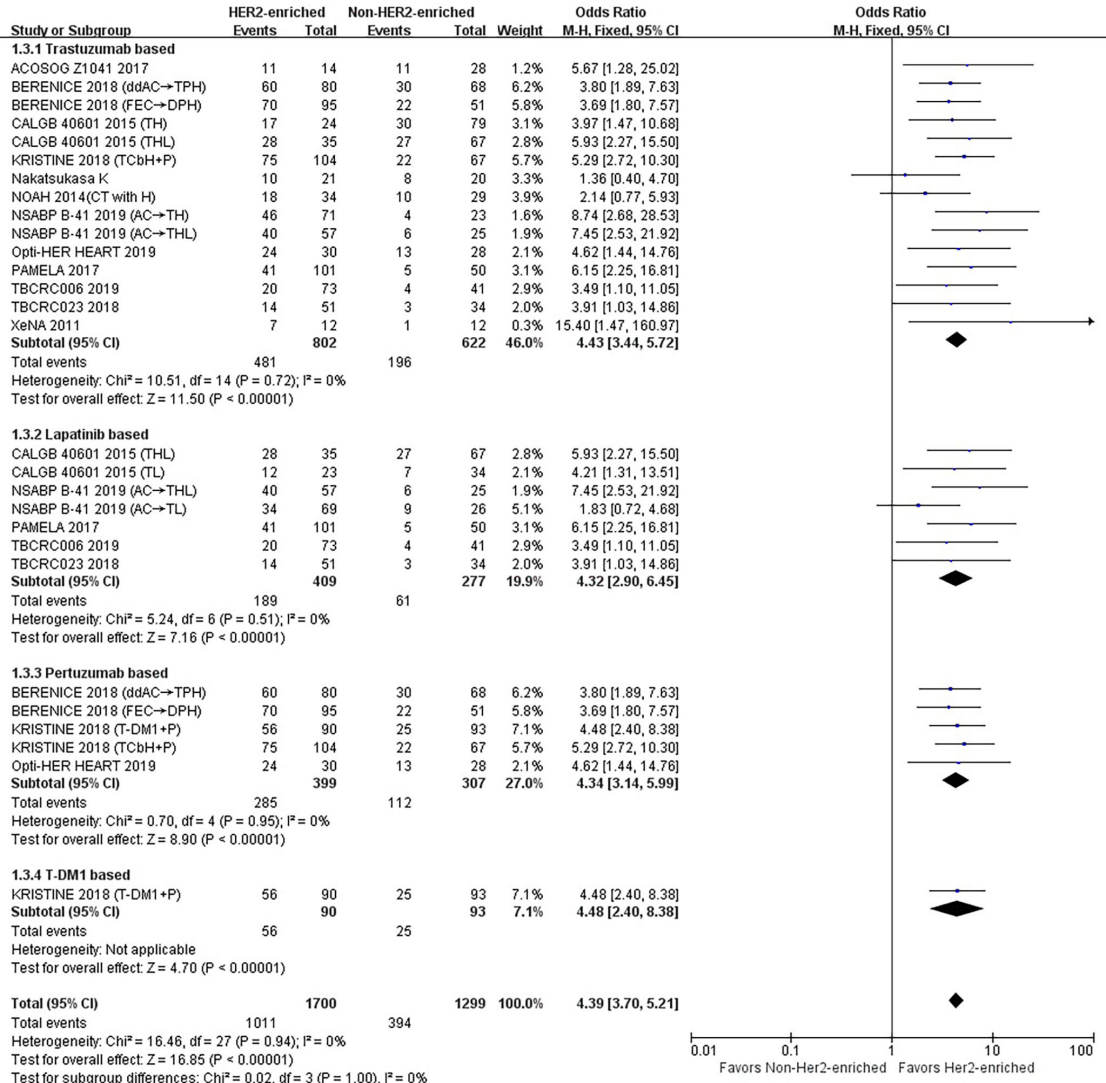
Forrest plot of odds ratio (OR) used to evaluate the correlations between intrinsic molecular subtype subgroups (HER2-enriched *vs.* non-HER2-enriched) and pCR as based on the category of HER2-targeted therapy used. CI, confidence interval; HER2, human epithelial growth factor receptor 2; pCR, pathological complete response.

The results showed that the pCR rate of trastuzumab treatment in HER2-enriched group was significantly improved compared with non-HER2-enriched subtype group (OR = 4.43, 95% CI = 3.44 to 5.72, *P* < 0.001), (*I* ^2^ = 0%, *P* = 0.72) (**Figure 4**).

#### Lapatinib

Seven studies or subgroups compared the correlations between the HER2-enriched subtype and pCR in trials including those treated with the HER2-targeted agent lapatinib (8, 22, 23, 27, 29). These studies included 409 patients belonging to the HER2-enriched and 277 non-HER2-enriched subtypes, with 189 and 61 patients exhibiting pCR, respectively. Five (22, 27, 29) and two (8, 23) of these studies utilized either neoadjuvant lapatinib and trastuzumab as dual HER2-targeted therapy or neoadjuvant lapatinib alone, respectively. Two studies used anthracyclines plus taxanes or in combination with other chemotherapeutics (23), and two used taxanes alone as chemotherapy regimens (8). Additionally, three studies used letrozole or TAM as endocrine treatment (22, 27, 29).

The results indicated that the pCR rate of lapatinib treatment in the HER2-enriched group was significantly improved *versus* non-HER2-enriched subtype group (OR = 4.32, 95% CI = 2.90 to 6.45, *P* < 0.001), (*I* ^2^ = 0%, *P* = 0.51) (**Figure 4**).

#### Pertuzumab

Five studies or subgroups estimated correlations between the HER2-enriched subtype and pCR in trials involving treatment with the HER2-targeted agent pertuzumab (20, 25, 28). These studies included 399 HER2-enriched and 307 non-HER2-enriched patients, with 285 and 112 patients exhibiting pCR, respectively. Four of these studies employed the neoadjuvant pertuzumab plus trastuzumab (20, 25), while one used pertuzumab plus T-DM1 (28) as dual HER2-targeted therapy. Three studies administered anthracyclines plus taxanes or combined with other chemotherapeutics (20, 25) and one study used only taxanes as chemotherapy regimens (28). Additionally, one study used only HER2-targeted therapy (28).

The results showed that the pCR rate of pertuzumab treatment within HER2-positive breast cancer cases in the HER2-enriched subtype group was obviously higher compared with the non-HER2-enriched subtype group (OR = 4.34, 95% CI = 3.14 to 5.99, *P* < 0.001), (*I* ^2^ = 0%, *P* = 0.95) (**Figure 4**).

#### T-DM1

There was only one study that assessed the relationship between the HER2-enriched subtype and pCR in a trial involving treatment with T-DM1 and HER2-targeted-based therapy (28). This included 90 HER2-enriched and 93 non-HER2-enriched patients, with 56 and 25 patients exhibiting pCR, respectively. This study employed neoadjuvant T-DM1 plus pertuzumab as dual HER2-targeted therapy, with no other therapies included.

The results indicated that the HER2-enriched subtype predicted a higher pCR rate than the non-HER2-enriched subtype involving T-DM1-based treatment (OR = 4.48, 95% CI = 2.40 to 8.38, *P* < 0.001) (**Figure 4**).

#### Trastuzumab Only- or Lapatinib Only-Based Chemotherapy

The pCR rate of either trastuzumab or lapatinib alone in addition to chemotherapy was reported in six (8, 11, 21, 23, 24, 26) and two studies (8, 23), respectively.

Three studies administered anthracyclines plus taxanes or in combination with other chemotherapeutics (11, 21, 23), and three used taxanes only as chemotherapy regimens for trastuzumab only-based treatment (8, 24, 26). For the lapatinib only-based treatment either anthracyclines plus taxanes in combination with cyclophosphamide were applied (23) or only taxanes were used as chemotherapy regimens within each study (8).

The results showed that the HER2-enriched subtype was correlated with higher pCR both for trastuzumab (OR = 3.80, 95% CI = 2.34 to 6.17, *P* < 0.001) and lapatinib (OR = 2.52, 95% CI = 1.22 to 5.22, *P* < 0.001) treatments compared to the non-HER2-enriched subtype. Low heterogeneity was found among the included individual studies (*I* ^2^ = 33%, *P* = 0.19; *I* ^2^ = 16%, *P* = 0.28, respectively) (**Supplementary Figure S2**).

### Correlations Between HER2-Enriched Subtype and pCR Rates According to the Neoadjuvant Chemotherapy Regimen

#### Taxanes

Six studies or subgroups estimated the correlations between taxane-based chemotherapy and pCR in trials involving HER2-targeted therapy (8, 24, 26, 28). These studies included 219 HER2-enriched and 279 non-HER2-enriched subtype patients, with 149 and 95 patients exhibiting pCR, respectively.

Three of these studies used taxanes as a single chemotherapy agent (8), while the other three used taxanes combined with other chemotherapy agents (24, 26, 28). Three studies (8, 24, 26) and one (8) study administered either trastuzumab or lapatinib as single HER2-targeted therapies, respectively. Individual studies administered either trastuzumab plus lapatinib (8) or trastuzumab plus pertuzumab (28) as dual HER2-targeted therapies.

**Figure 5:**
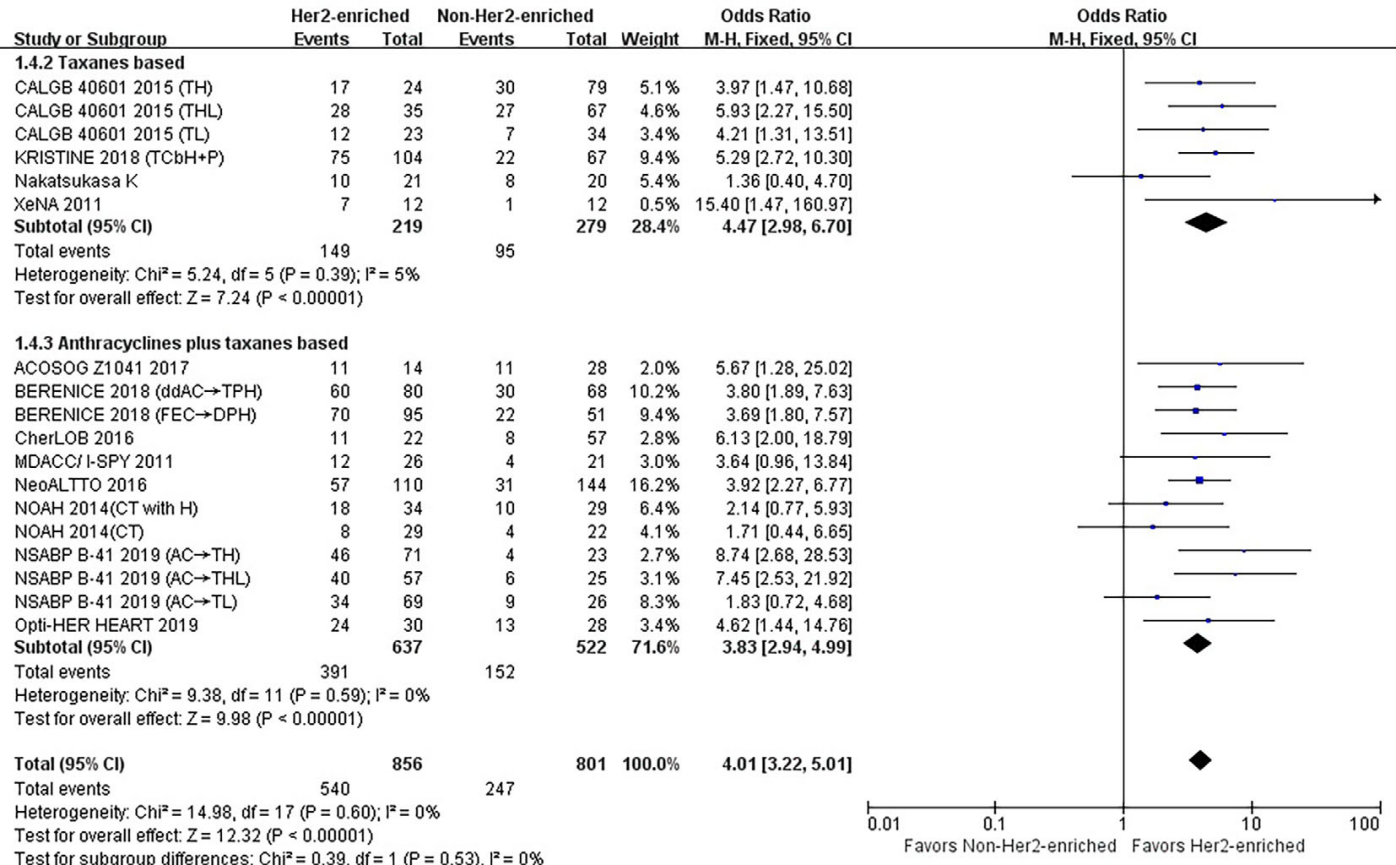
Forrest plot of odds ratio (OR) used to evaluate the correlations between intrinsic molecular subtype subgroups (HER2-enriched *vs.* non-HER2-enriched) and pCR as based on the type of chemotherapy used. CI, confidence interval; HER2, human epithelial growth factor receptor 2; pCR, pathological complete response.

The results showed that the pCR rate for taxane treatment in HER2-enriched subtype group was substantially higher than in non-HER2-enriched subtype group (OR = 4.47, 95%CI = 2.98 to 6.70, *P* < 0.001), (*I* ^2^ = 5%, *P* = 0.39) (**Figure 5**).

#### Anthracyclines Plus Taxanes

Twelve studies or subgroups estimated correlations between the HER2-enriched subtype and pCR in trials involving chemotherapy with anthracyclines plus taxanes (10–12, 20, 21, 23, 25, 26). These studies included 637 HER2-enriched and 522 non-HER2-enriched patients, with 391 and 152 patients exhibiting pCR, respectively.

Three (11, 21, 23) and one (23) of these studies used either the neoadjuvant trastuzumab or lapatinib as single HER2-targeted therapies, respectively, while one (23) and three (20, 25) employed trastuzumab plus lapatinib or trastuzumab plus pertuzumab as dual HER2-targeted therapies, respectively. Two studies administered trastuzumab, lapatinib, or a combination of the two as HER2-targeted therapy (10, 12), while two studies used only chemotherapy (11, 26).

The results showed that the pCR rate of anthracyclines plus taxanes treatment for HER2-positive breast cancer patients was significantly improved in HER2-enriched subtype group (OR = 3.83, 95% CI = 2.94 to 4.99, *P* < 0.001), (*I* ^2^ = 0%, *P* = 0.59) (**Figure 5**).

### Correlations Between the HER2-Enriched Subtype and pCR Rates According to Neoadjuvant Endocrine Therapy

There were included three trials that evaluated correlations between the HER2-enriched subtype and pCR in those treated with letrozole or TAM-based endocrine therapy (22, 27, 29). These studies included 225 HER2-enriched and 125 non-HER2-enriched subtype patients, with 75 and 12 patients exhibiting pCR, respectively. All of these studies utilized trastuzumab plus lapatinib as dual HER2-targeted therapy.

**Figure 6:**
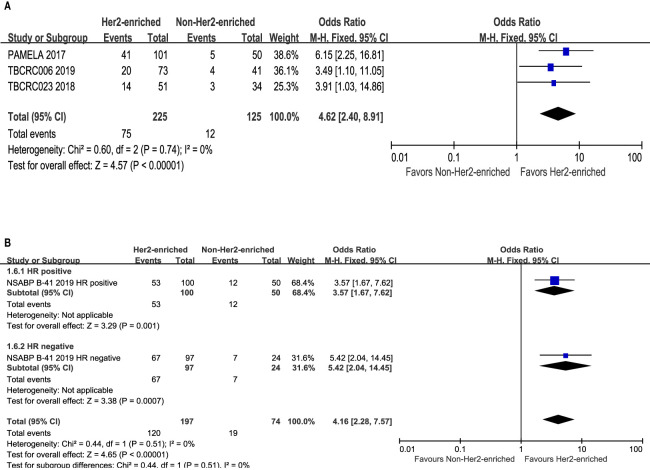
Forrest plot of odds ratio (OR) used to evaluate the correlations between nintrinsic molecular subtype subgroups (HER2-enriched *vs.* non-HER2-enriched) and pCR as based on: **(A)** endocrine therapy used; **(B)** hormone receptors status. CI, confidence interval; HER2, human epithelial growth factor receptor 2; pCR, pathological complete response.

The results showed that the pCR rate for patients with HER2-positive breast cancer belonging to the HER2-enriched subtype was obviously higher than that for those within the non-HER2-enriched subtype (OR = 4.62, 95% CI = 2.40 to 8.91, *P* < 0.001). Low heterogeneity was observed (*I* ^2^ = 0%, *P* = 0.74) among the included individual studies (**Figure 6A**).

### Correlations Between HER2-Enriched Subtype and pCR Rates According to the Hormone Receptor

Only one study provided detailed information concerning hormone receptor status and the pCR rate within the HER2-enriched and non-HER2-enriched subgroups (23). There were 100 or 97, and 50 or 24 hormone receptor-positive and -negative patients within the HER2-enriched and non-HER2-enriched subtype groups, respectively. Meanwhile, 53 and 67, or 12 and 7 patients achieved pCR, respectively.

The results showed that the pCR rates for either hormone receptor positive- or negative-HER2-positive breast cancer was significantly higher in the HER2-enriched subtype (OR = 3.57, 95% CI = 1.67 to 7.62, *P*= 0.001; OR = 5.42, 95% CI = 2.04 to 14.45, *P* < 0.001) compared to the non-HER2-enriched subtype. Low heterogeneity was observed (I^2^ = 0%, *P* = 0.51) among the included individual studies (**Figure 6B**).

### Correlations Between the HER2-Enriched Subtype and pCR Rates in Overall Patients Excluding Non-Randomized Studies

We further performed a sensitivity analysis excluding the seven non-randomized studies (20, 22, 24–27). However, the results showed that the overall effect was not significantly changed. The HER2-enriched type was correlated to a significantly higher pCR rate (OR= 4.23, 95% CI 3.32-5.38; *P* < 0.001) in patients overall, with low heterogeneity observed (I^2^ = 0%; *P* = 0.64) (**Supplementary Figure 3**).

## Discussion

To best of our knowledge, this is most recent and comprehensive meta-analysis including prospective studies evaluating the association between an HER2-enriched subtype and pCR rates in HER2-positive breast cancer patients receiving neoadjuvant treatment. The results indicated that an HER2-enriched subtype reliably predicted the pCR rate following treatment. Its predictive role shows specificity for HER2-targeted agent-based treatments, as it was not correlated to the pCR rate in patients undergoing treatment with HER2-targeted-free agents. In addition, this association existed in every subgroup regardless of whether the patients received neoadjuvant treatment, as analyzed by the number (single or dual) of HER2-targeted agents used, the category of HER2-targeted agents, neoadjuvant chemotherapy regimen and neoadjuvant endocrine therapy. In addition, the HER2-enriched subtype was correlated with the pCR rates regardless of hormone receptor status.

Neoadjuvant treatment is being increasingly administrated for HER2-positive breast cancer to downstage tumors, increase resectability and the possibility of breast conservation (1, 30). Moreover, pCR within HER2-positive breast cancer is related to significantly improve long-term outcomes (5, 30). In adjuvant settings, the KATHERINE trial also showed that HER2-positive breast cancer patients who did not achieve pCR after neoadjuvant treatment could substantially benefit from intensified TDM1 treatment *via* a 50% reduction in risk of recurrence of invasive breast cancer or post-operative death (31). Therefore, it became vitally important and useful for clinicians to screen those with sensitive HER2-positive breast cancer who might achieve pCR following neoadjuvant treatment, either if they are treated in a neoadjuvant or adjuvant setting. Some studies have investigated certain biomarkers that might guide such individualized therapies. For example, the infiltration of TILs or *PIK3CA* mutations were associated with the pCR rate in NeoALTTO, CherLOB, NeoSphere, TBCRC006, GeparQuattro, GeparQuinto, and GeparSixto trials (9, 32–40).

As a subtype of intrinsic molecular classification, HER2-enriched tissues have high expression of HER2-regulated and proliferation-related genes but low expression of luminal-related genes. Such extensive activation of the HER2/EGFR signaling pathway indicates that tumor cells depend on the HER2 receptor within the HER2-enriched subtype, and thus these patients may benefit the most from HER2-targeted treatment (2, 9, 41). However, a recent meta-analysis showed HER2-enriched subtype was related to pCR in all patients, while it seems that this meta-analysis could not distinguish the different Her2-targeted agents (42). Our study differs from that of Schettini F et al. in the secondary outcome section, and we analyzed the relationship between HER2-enriched subtypes and pCR rates according to the number and class of HER2-targeting drugs employed, and our study is a necessary complement to the study of Schettini F et al. Our meta-analysis was based on prospective studies and confirms that the HER2-enriched subtype plays a relevant role in defining the possibility of increased pCR; moreover, we analyzed the number of HER2-targeted-based agents and the category of HER2-targeted-based agents.

We further estimated whether the roles of the HER2-enriched subtype in prediction of pCR for HER2-positive breast cancer varied among different treatment options, including the number and category of HER2 agents, chemotherapy regimens, endocrine therapy, as well as hormone receptor status. HER2-target agent-based therapy, especially when combined with chemotherapy, has been the standard neoadjuvant treatment for HER2-positive breast cancer. The results of this meta-analysis demonstrated that an HER2-enriched subtype can predict the pCR of HER2-target agent-based therapy with either single or dual HER2-target agent combined regimens. Nevertheless, the HER2-enriched subtype appears not to predict pCR with chemotherapy consisting of HER2-target-free agents. This also suggests that the HER2-enriched subtype is a specificity biomarker for HER2-target agent-based treatments. Interestingly, such roles were not demonstrated by other meta-analyses focusing on *PIK3CA* mutations, phosphatase and tensin homolog loss, and TILs (32, 38, 43).

The predictive value of markers might be related to a different mechanism of HER2-targeted agents. For instance, *PIK3CA* mutations could only predicted the pCR rate after single-targeting trastuzumab treatment, but not following single-targeting lapatinib treatment within another meta-analysis (44). HER2-targeted agents within the studies in this meta-analysis included trastuzumab, pertuzumab, lapatinib, and T-DM1. These drugs target the HER2 oncogene differently, though all can inhibit the HER2 signal pathway. Trastuzumab and pertuzumab are monoclonal antibodies that bind to the extracellular domain of HER2; lapatinib belongs to the tyrosine kinase inhibitors, which compete with the ATP binding site of the catalytic domain of HERs, and T-DM1 is an antibody drug conjugate (45–49). Our meta-analysis showed that the HER2-enriched subtype consistently predicts the pCR of HER2-target agents despite their categories and subtle differences of targeting HER2.

Despite multiple frequencies, dosing, sequencing, and combination with HER2-target agents, the chemotherapy regimens of most studies included in this meta-analysis contained anthracycline and/or taxanes. The results indicated that the roles of the HER2-enriched subtype in predicting pCR for HER2-positive breast cancer patients are independent of anthracycline-based, taxane-based or the combination therapy of the two, even though these chemotherapeutics have different pharmacological mechanisms. It also implies that as a predictor, the HER2-enriched subtype is likely to guide either anthracycline-based or taxane-based chemotherapy combined with HER2-targeted agents. Two meta-analyses demonstrated that the predictive role of pCR for TIL and *PIK3CA* mutations were also independent of the chemotherapy drugs used for treating HER2-positive breast cancer (32, 38).

Predictors for HER2-positive breast cancer in those receiving HER2-targeted agent-based neoadjuvant endocrine therapy were seldom reported. Our pooled analysis of three studies that included a relatively large combined sample size (350 patients) indicated that the HER2-enriched subtype might also be an effective predictor of pCR in HER2-target agent-based neoadjuvant endocrine therapy. The most recent study suggested that patients with high *HER2* amplification and an intact PI3K pathway were substantially sensitive to HER2-targeted therapies either with or without endocrine therapy in a TBCRC006 trial (39).

We also found that the pCR rate within the HER2-enriched subtype was significantly higher than that within the non-HER2-enriched subtype, while it should be noted that the latter category contained only a limited number of patients. Nevertheless, this meta-analysis found that the HER2-enriched subtype had a similar predictive value for indicating pCR both in hormone receptor-negative and -positive patients. This suggests that the predictive role of the HER2-enriched subtype is not influenced by hormone receptor status. Of note, the predictive roles of the HER2-enriched subtype differed with the *PIK3CA* mutant, which displayed a significant interaction with the hormone receptor for treatment response (40, 44). A pooled analysis of five trials found that within patients containing a *PIK3CA* mutation, those that were hormone-receptor positive had a significantly poorer pCR rate (7.6%) than those belonging to the hormone-receptor negative group (27.2%) (40). Nevertheless, the limited number of samples contained within this meta-analysis and the bidirectional crosstalk between HER2 and the hormone-receptor pathways in resistance to HER2-targeted and endocrine therapy should not be ignored (48).

Some limitations of the present meta-analysis should be noted in the interpretation of the results. First, our study was based on aggregated data from published literature rather than from individual patients; therefore, the relationship between the HER2-enriched subtype and pCR with other important clinicopathological parameters—including age of patient, exact stage of subgroup, and duration of treatment—was not possible to estimate. Second, differing definitions of pCR in studies (ypT0/is or ypT0/is ypN0) might also have some influence on the reliability of the results. Third, this meta-analysis also included seven non-randomized studies, although all of these studies were prospective. However, we have formulated strict inclusion and exclusion standards, and the included data were from the actual treatment and pathological results of studies. In addition, the meta-analysis included a large sample size (N = 2,190) from 14 studies with the pooled analyses exhibiting no significant heterogeneity. We uniformly adopted the definition of pCR for primary objectives in studies according to the American Joint Committee on Cancer Staging Manual, 7th edition (49). Sensitivity analyses found the results to be unchanged after excluding non-randomized studies. Therefore, these limitations should not influence the overall interpretation of the results due to the strict methodology used.

## Conclusion

This meta-analysis shows a significant correlation between the HER2-enriched subtype at baseline and clinical treatment outcomes in HER-2-positive breast cancer patients. An HER2-enriched subtype may serve as a robust marker for predicting the pCR rate within patients receiving HER2-targeted agent-based neoadjuvant treatment. This could play important predictive roles within regimens in which either single or dual HER2 blockade, HER2-targeted agent combined chemotherapy or endocrine therapy is employed, and for hormone receptor status. Moreover, it appears that HER2 enrichment is a specific predictor for HER2-targeted agent-based therapy. Novel trial designs concerning biomarker research including the HER2-enriched subtype are urgently needed in the future.

## Data Availability Statement

The raw data supporting the conclusions of this article will be made available by the authors, without undue reservation.

## Author Contributions

GS, JZ, FuZ, and XH contributed to the conception and the drafting of manuscripts. DR, FD, and FaZ are responsible for coordinating and participating in the article revision. All authors contributed to the article and approved the submitted version.

## Funding

This work was supported by grants from the Thousand Talents of Program of High-end Innovation of Qinghai Province in China (for JZ). The sponsors played no role in the study design, data collection, analysis, or decision to submit the article for publication.

## Conflict of Interest

The authors declare that the research was conducted in the absence of any commercial or financial relationships that could be construed as a potential conflict of interest.

## Supplementary Material

Supplementary Figure 1 Flow chart for the selection process of eligible publications.

Supplementary Figure 2 Forrest plot of odds ratio (OR) used to evaluate the correlations between intrinsic molecular subtype subgroups (HER2-enriched *vs.* non-HER2-enriched) and pCR as based on trastuzumab only- or lapatinib only-based chemotherapy. CI, confidence interval; HER2, human epithelial growth factor receptor 2; pCR, pathological complete response.

Supplementary Figure 3 Forrest plot of odds ratio (OR) used to evaluate the correlations between intrinsic molecular subtype subgroups (HER2-enriched *vs.* non-HER2-enriched) and pCR in overall patients excluding non-randomized studies. CI, confidence interval; HER2, human epithelial growth factor receptor 2; pCR, pathological complete response.

## References

[1] Harbeck N Gnant M. Breast Cancer. Lancet (2017) 389(10074):1134–50. 10.1016/S0140-6736(16)31891-8 27865536

[2] Cancer Genome Atlas Network. Comprehensive Molecular Portraits of Human Breast Tumours. Nature (2012) 490(7418):61–70. 10.1038/nature11412 23000897 PMC3465532

[3] Bonnefoi H Litière S Piccart M MacGrogan G Fumoleau P Brain E. Pathological Complete Response After Neoadjuvant Chemotherapy is an Independent Predictive Factor Irrespective of Simplified Breast Cancer Intrinsic Subtypes: A Landmark and Two-Step Approach Analyses From the EORTC 10994/BIG 1-00 Phase III Trial. Ann Oncol (2014) 25(6):1128–36. 10.1093/annonc/mdu118 PMC4037859 24618153

[4] Wuerstlein R Harbeck N. Neoadjuvant Therapy for HER2-Positive Breast Cancer. Rev Recent Clin Trials (2017) 12(2):81–92. 10.2174/1574887112666170202165049 28164759

[5] Broglio KR Quintana M Foster M Olinger M McGlothlin A Berry SM. Association of Pathologic Complete Response to Neoadjuvant Therapy in HER2-Positive Breast Cancer With Long-Term Outcomes: A Meta-Analysis. JAMA Oncol (2016) 2(6):751–60. 10.1001/jamaoncol.2015.6113 26914222

[6] Baselga J Bradbury I Eidtmann H Di Cosimo S de Azambuja E Aura C. Lapatinib With Trastuzumab for HER2-Positive Early Breast Cancer (NeoALTTO): A Randomised, Open-Label, Multicentre, Phase 3 Trial. Lancet (2012) 379(9816):633–40. 10.1016/S0140-6736(11)61847-3 PMC5705192 22257673

[7] Robidoux A Tang G Rastogi P Geyer CEJ Azar CA Atkins JN. Lapatinib as a Component of Neoadjuvant Therapy for HER2-Positive Operable Breast Cancer (NSABP Protocol B-41): An Open-Label, Randomised Phase 3 Trial. Lancet Oncol (2013) 14(12):1183–92. 10.1016/S1470-2045(13)70411-X 24095300

[8] Carey LA Berry DA Cirrincione CT Barry WT Pitcher BN Harris LN. Molecular Heterogeneity and Response to Neoadjuvant Human Epidermal Growth Factor Receptor 2 Targeting in CALGB 40601, a Randomized Phase III Trial of Paclitaxel Plus Trastuzumab With or Without Lapatinib. J Clin Oncol (2016) 34(6):542–9. 10.1200/JCO.2015.62.1268 PMC4980567 26527775

[9] Di Modica M Tagliabue E Triulzi T. Predicting the Efficacy of HER2-Targeted Therapies: A Look at the Host. Dis Markers (2017) 2017:7849108. 10.1155/2017/7849108 29403144 PMC5748305

[10] Fumagalli D Venet D Ignatiadis M Azim HAJ Maetens M Rothé F. RNA Sequencing to Predict Response to Neoadjuvant Anti-HER2 Therapy: A Secondary Analysis of the NeoALTTO Randomized Clinical Trial. JAMA Oncol (2017) 3(2):227–34. 10.1001/jamaoncol.2016.3824 PMC5374044 27684533

[11] Prat A Bianchini G Thomas M Belousov A Cheang MCU Koehler A. Research-Based PAM50 Subtype Predictor Identifies Higher Responses and Improved Survival Outcomes in HER2-Positive Breast Cancer in the NOAH Study. Clin Cancer Res (2014) 20(2):511–21. 10.1158/1078-0432.CCR-13-0239 24443618

[12] Dieci MV Prat A Tagliafico E Paré L Ficarra G Bisagni G. Integrated Evaluation of PAM50 Subtypes and Immune Modulation of pCR in HER2-Positive Breast Cancer Patients Treated With Chemotherapy and HER2-Targeted Agents in the CherLOB Trial. Ann Oncol (2016) 27(10):1867–73. 10.1093/annonc/mdw262 27484801

[13] Prat A Pascual T Adamo B. Intrinsic Molecular Subtypes of HER2+ Breast Cancer. Oncotarget (2017) 8(43):73362–3. 10.18632/oncotarget.20629 PMC5650264 29088709

[14] Parker JS Mullins M Cheang MCU Leung S Voduc D Vickery T. Supervised Risk Predictor of Breast Cancer Based on Intrinsic Subtypes. J Clin Oncol (2009) 27(8):1160–7. 10.1200/JCO.2008.18.1370 PMC2667820 19204204

[15] Knobloch K Yoon U Vogt PM. Preferred Reporting Items for Systematic Reviews and Meta-Analyses (PRISMA) Statement and Publication Bias. J Craniomaxillofac Surg (2011) 39(2):91–2. 10.1016/j.jcms.2010.11.001 21145753

[16] Green S Higgins JP Alderson P Clarke M Mulrow CD Oxman AD. Cochrane Handbook for Systematic Reviews of Interventions: Cochrane Book Series. Chichester, UK John Wiley & Sons, Ltd (2008). Version 5.1.0.

[17] DerSimonian R Laird N. Meta-Analysis in Clinical Trials. Control Clin Trials (1986) 7(3):177–88. 10.1016/0197-2456(86)90046-2 3802833

[18] Higgins JP Altman DG Gøtzsche PC Jüni P Moher D Oxman AD. The Cochrane Collaboration’s Tool for Assessing Risk of Bias in Randomised Trials. BMJ (2011) 343:d5928. 10.1136/bmj.d5928 22008217 PMC3196245

[19] Egger M Davey Smith G Schneider M Minder C. Bias in Meta-Analysis Detected by a Simple, Graphical Test. BMJ (1997) 315(7109):629–34. 10.1136/bmj.315.7109.629 PMC2127453 9310563

[20] Swain SM Ewer MS Viale G Delaloge S Ferrero J-M Verrill M. Pertuzumab, Trastuzumab, and Standard Anthracycline- and Taxane-Based Chemotherapy for the Neoadjuvant Treatment of Patients With HER2-Positive Localized Breast Cancer (BERENICE): A Phase II, Open-Label, Multicenter, Multinational Cardiac Safety Study. Ann Oncol (2018) 29(3):646–53. 10.1093/annonc/mdx773 PMC5888999 29253081

[21] Lesurf R Griffith OL Griffith M Hundal J Trani L Watson MA. Genomic Characterization of HER2-Positive Breast Cancer and Response to Neoadjuvant Trastuzumab and Chemotherapy-Results From the ACOSOG Z1041 (Alliance) Trial. Ann Oncol (2017) 28(5):1070–7. 10.1093/annonc/mdx048 PMC5790063 28453704

[22] Llombart-Cussac A Cortés J Paré L Galván P Bermejo B Martínez N. HER2-Enriched Subtype as a Predictor of Pathological Complete Response Following Trastuzumab and Lapatinib Without Chemotherapy in Early-Stage HER2-Positive Breast Cancer (PAMELA): An Open-Label, Single-Group, Multicentre, Phase 2 Trial. Lancet Oncol (2017) 18(4):545–54. 10.1016/S1470-2045(17)30021-9 28238593

[23] Swain SM Tang G Lucas PC Robidoux A Goerlitz D Harris BT. Pathologic Complete Response and Outcomes by Intrinsic Subtypes in NSABP B-41, a Randomized Neoadjuvant Trial of Chemotherapy With Trastuzumab, Lapatinib, or the Combination. Breast Cancer Res Treat (2019) 178(2):389–99. 10.1007/s10549-019-05398-3 PMC6797698 31428908

[24] Nakatsukasa K Koyama H Oouchi Y Imanishi S Mizuta N Sakaguchi K. Docetaxel, Cyclophosphamide, and Trastuzumab as Neoadjuvant Chemotherapy for HER2-Positive Primary Breast Cancer. Breast Cancer (2017) 24(1):92–7. 10.1007/s12282-016-0677-4 26874836

[25] Gavilá J Oliveira M Pascual T Perez-Garcia J Gonzàlez X Canes J. Safety, Activity, and Molecular Heterogeneity Following Neoadjuvant Non-Pegylated Liposomal Doxorubicin, Paclitaxel, Trastuzumab, and Pertuzumab in HER2-Positive Breast Cancer (Opti-HER HEART): An Open-Label, Single-Group, Multicenter, Phase 2 Trial. BMC Med (2019) 17(1):8. 10.1186/s12916-018-1233-1 30621698 PMC6325829

[26] Cheang MCU Prat A Fan C Perou CM. S5-2: PAM50 HER2– Enriched Subtype Enriches for Tumor Response to Neoadjuvant Anthracyclines/Taxane and Trastuzumab/Taxane Containing Regimens in HER2– Positive Breast Cancer. Cancer Res (2011) 71(24 Suppl). Abstract nr S5-2. 10.1158/0008-5472

[27] Prat A Pascual T De Angelis C Gutierrez C Llombart-Cussac A Wang T. HER2-Enriched Subtype and ERBB2 Expression in HER2-Positive Breast Cancer Treated With Dual HER2 Blockade. J Natl Cancer Inst (2020) 112:46–54. 10.1093/jnci/djz042 31037288 PMC7850037

[28] Prat A Slamon D Hurvitz SA Press MF Phillips GL Valverde VL. Abstract PD3-06: Association of Intrinsic Subtypes With Pathological Complete Response (pCR) in the KRISTINE Neoadjuvant Phase 3 Clinical Trial in HER2-Positive Early Breast Cancer (EBC). Cancer Res (2018) 78(4 Suppl). Abstract nr PD3-06. 10.1158/1538-7445

[29] Prat A De Angelis C Pascual T Gutierrez C Wang T Paré L. Abstract P2-09-12: Independent Validation of the HER2-Enriched Subtype as a Predictor of Pathological Complete Response Following Trastuzumab and Lapatinib Without Chemotherapy in Early-Stage HER2-Positive Breast Cancer. Cancer Res (2018) 78(4 Suppl). Abstract nr P2-09-12. 10.1158/1538-7445

[30] Pusztai L Foldi J Dhawan A DiGiovanna MP Mamounas EP. Changing Frameworks in Treatment Sequencing of Triple-Negative and HER2-Positive, Early-Stage Breast Cancers. Lancet Oncol (2019) 20(7):e390–6. 10.1016/S1470-2045(19)30158-5 31267973

[31] von Minckwitz G Huang CS Mano MS Loibl S Mamounas EP Untch M. Trastuzumab Emtansine for Residual Invasive HER2-Positive Breast Cancer. N Engl J Med (2019) 380(7):617–28. 10.1056/NEJMoa1814017 30516102

[32] Solinas C Ceppi M Lambertini M Scartozzi M Buisseret L Garaud S. Tumor-Infiltrating Lymphocytes in Patients With HER2-Positive Breast Cancer Treated With Neoadjuvant Chemotherapy Plus Trastuzumab, Lapatinib or Their Combination: A Meta-Analysis of Randomized Controlled Trials. Cancer Treat Rev (2017) 57:8–15. 10.1016/j.ctrv.2017.04.005 28525810

[33] Denkert C von Minckwitz G Brase JC Sinn BV Gade S Kronenwett R. Tumor-Infiltrating Lymphocytes and Response to Neoadjuvant Chemotherapy With or Without Carboplatin in Human Epidermal Growth Factor Receptor 2-Positive and Triple-Negative Primary Breast Cancers. J Clin Oncol (2015) 33(9):983–91. 10.1200/JCO.2014.58.1967 25534375

[34] Salgado R Denkert C Campbell C Savas P Nuciforo P Aura C. Tumor-Infiltrating Lymphocytes and Associations With Pathological Complete Response and Event-Free Survival in HER2-Positive Early-Stage Breast Cancer Treated With Lapatinib and Trastuzumab: A Secondary Analysis of the NeoALTTO Trial. JAMA Oncol (2015) 1(4):448–54. 10.1001/jamaoncol.2015.0830 PMC5551492 26181252

[35] Ingold Heppner B Untch M Denkert C Pfitzner BM Lederer B Schmitt W. Tumor-Infiltrating Lymphocytes: A Predictive and Prognostic Biomarker in Neoadjuvant-Treated HER2-Positive Breast Cancer. Clin Cancer Res (2016) 22(23):5747–54. 10.1158/1078-0432.CCR-15-2338 27189162

[36] Ignatiadis M Van den Eynden G Roberto S Fornili M Bareche Y Desmedt C. Tumor-Infiltrating Lymphocytes in Patients Receiving Trastuzumab/Pertuzumab-Based Chemotherapy: A TRYPHAENA Substudy. J Natl Cancer Inst (2019) 111(1):69–77. 10.1093/jnci/djy076 29788230 PMC6335115

[37] Loibl S von Minckwitz G Schneeweiss A Paepke S Lehmann A Rezai M. PIK3CA Mutations Are Associated With Lower Rates of Pathologic Complete Response to Anti-Human Epidermal Growth Factor Receptor 2 (Her2) Therapy in Primary HER2-Overexpressing Breast Cancer. J Clin Oncol (2014) 32(29):3212–20. 10.1200/JCO.2014.55.7876 25199759

[38] Ibrahim EM Kazkaz GA Al-Mansour MM Al-Foheidi ME. The Predictive and Prognostic Role of Phosphatase Phosphoinositol-3 (PI3) Kinase (PIK3CA) Mutation in HER2-Positive Breast Cancer Receiving HER2-Targeted Therapy: A Meta-Analysis. Breast Cancer Res Treat (2015) 152(3):463–76. 10.1007/s10549-015-3480-6 26105797

[39] Veeraraghavan J De Angelis C Mao R Wang T Herrera S Pavlick AC. A Combinatorial Biomarker Predicts Pathologic Complete Response to Neoadjuvant Lapatinib and Trastuzumab Without Chemotherapy in Patients With HER2+ Breast Cancer. Ann Oncol (2019) 30:927–33. 10.1093/annonc/mdz076 PMC6594453 30903140

[40] Loibl S Majewski I Guarneri V Nekljudova V Holmes E Bria E. PIK3CA Mutations are Associated With Reduced Pathological Complete Response Rates in Primary HER2-Positive Breast Cancer: Pooled Analysis of 967 Patients From Five Prospective Trials Investigating Lapatinib and Trastuzumab. Ann Oncol (2016) 27(8):1519–25. 10.1093/annonc/mdw197 PMC6279074 27177864

[41] Bartlett JM McConkey CC Munro AF Desmedt C Dunn JA Larsimont DP. Predicting Anthracycline Benefit: TOP2A and CEP17-Not Only But Also. J Clin Oncol (2015) 33(15):1680–7. 10.1200/JCO.2013.54.7869 25897160

[42] Schettini F Pascual T Conte B Chic N Brasó-Maristany F Galván P. HER2-Enriched Subtype and Pathological Complete Response in HER2-Positive Breast Cancer: A Systematic Review and Meta-Analysis. Cancer Treat Rev (2020) 84:101965. 10.1016/j.ctrv.2020.101965 32000054 PMC7230134

[43] Wang Y Liu Y Du Y Yin W Lu J. The Predictive Role of Phosphatase and Tensin Homolog (PTEN) Loss, Phosphoinositol-3 (PI3) Kinase (PIK3CA) Mutation, and PI3K Pathway Activation in Sensitivity to Trastuzumab in HER2-Positive Breast Cancer: A Meta-Analysis. Curr Med Res Opin (2013) 29(6):633–42. 10.1185/03007995.2013.794775 23574264

[44] Fan H Li C Xiang Q Xu L Zhang Z Liu Q. PIK3CA Mutations and Their Response to Neoadjuvant Treatment in Early Breast Cancer: A Systematic Review and Meta-Analysis. Thorac Cancer (2018) 9(5):571–9. 10.1111/1759-7714.12618 PMC5928352 29575819

[45] Moasser MM Krop IE. The Evolving Landscape of HER2 Targeting in Breast Cancer. JAMA Oncol (2015) 1(8):1154–61. 10.1001/jamaoncol.2015.2286 26204261

[46] Oh DY Bang YJ. HER2-Targeted Therapies - a Role Beyond Breast Cancer. Nat Rev Clin Oncol (2020) 17(1):33–48. 10.1038/s41571-019-0268-3 31548601

[47] Gomez-Martín C Lopez-Rios F Aparicio J Barriuso J García-Carbonero R Pazo R. A Critical Review of HER2-Positive Gastric Cancer Evaluation and Treatment: From Trastuzumab, and Beyond. Cancer Lett (2014) 351(1):30–40. 10.1016/j.canlet.2014.05.019 24943493

[48] Montemurro F Di Cosimo S Arpino G. Human Epidermal Growth Factor Receptor 2 (HER2)-Positive and Hormone Receptor-Positive Breast Cancer: New Insights Into Molecular Interactions and Clinical Implications. Ann Oncol (2013) 24(11):2715–24. 10.1093/annonc/mdt287 23908178

[49] Edge S Byrd D Compton C Fritz A Greene F Trotti A. Purposes and Principles of Cancer Staging. In: Edge S Byrd DR Compton CC Fritz AG Greene FL Trotti A, editors. AJCC Cancer Staging Manual, 7th edition. New York, NY: Springer-Verlag (2010). p. 3–15.

