# Supplementary figures and images for: Meta-Analysis of HER2-Enriched Subtype Predicting the Pathological Complete Response Within HER2-Positive Breast Cancer in Patients Who Received Neoadjuvant Treatment
Source: Front Oncol. 2021 Jul 23;11:632357. doi: 10.3389/fonc.2021.632357 (PMC8343531; doi:10.3389/fonc.2021.632357)

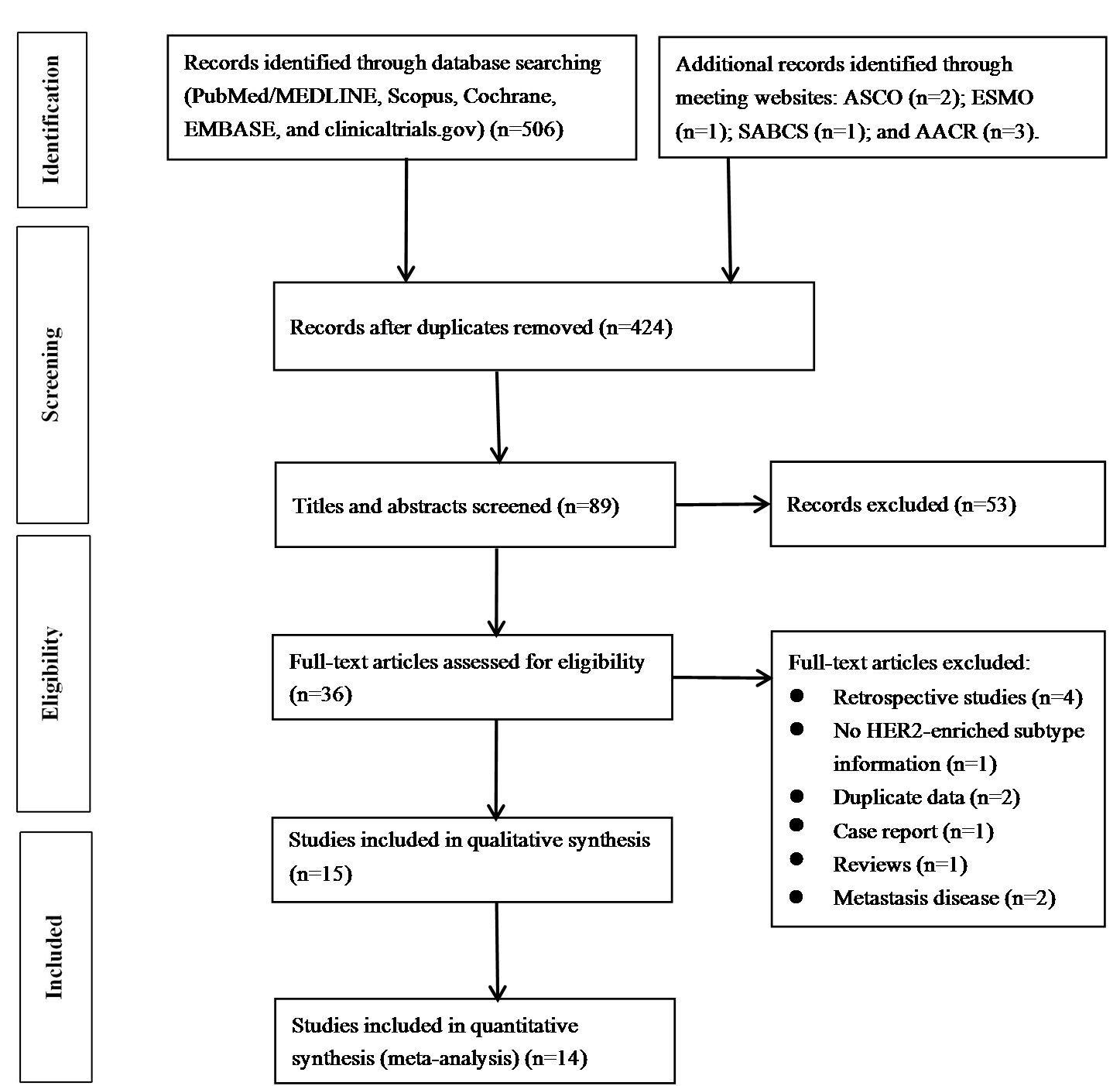

Supplement: Supplementary Figure 1 — Flow chart for the selection process of eligible publications. [file Image_1.jpeg]

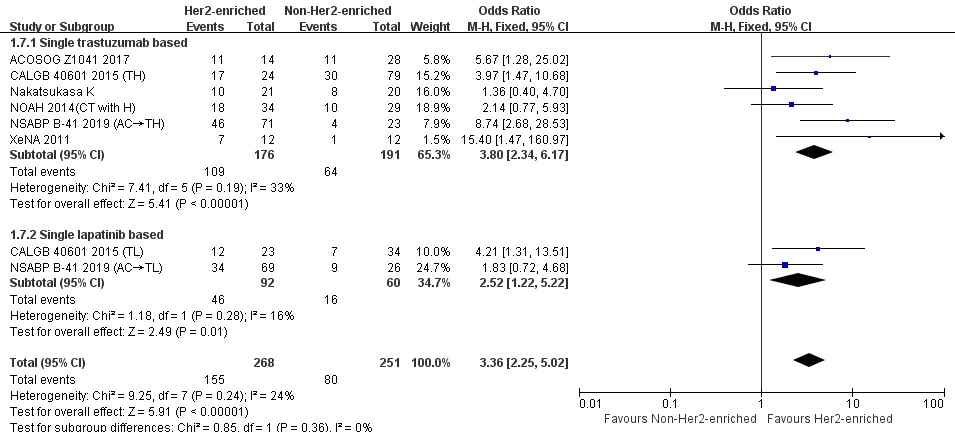

Supplement: Supplementary Figure 2 — Forrest plot of odds ratio (OR) used to evaluate the correlations between intrinsic molecular subtype subgroups (HER2-enriched vs. non-HER2-enriched) and pCR as based on trastuzumab only- or lapatinib only-based chemotherapy. CI, confidence interval; HER2, human epithelial growth factor receptor 2; pCR, pathological complete response. [file Image_2.jpeg]

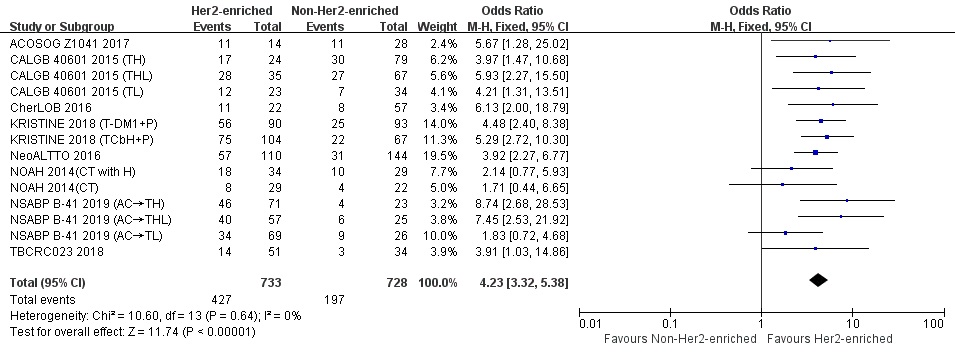

Supplement: Supplementary Figure 3 — Forrest plot of odds ratio (OR) used to evaluate the correlations between intrinsic molecular subtype subgroups (HER2-enriched vs. non-HER2-enriched) and pCR in overall patients excluding non-randomized studies. CI, confidence interval; HER2, human epithelial growth factor receptor 2; pCR, pathological complete response. [file Image_3.jpeg]
